# Right ventricular function impaired in children and adolescents with severe idiopathic scoliosis

**DOI:** 10.1186/1748-7161-8-1

**Published:** 2013-01-14

**Authors:** Shujuan Li, Junlin Yang, Yunquan Li, Ling Zhu, Yuese Lin, Xuandi Li, Zifang Huang, Huishen Wang

**Affiliations:** 1 Department of Paediatric Cardiology, the First Affiliated Hospital of Sun Yat-sen University, No.58 2nd Zhongshan Road, Guangzhou 510080, China; 2 Department of Spinal Surgery, the First Affiliated Hospital of Sun Yat-sen University, No.58 2nd Zhongshan Road, Guangzhou, 510080, China

**Keywords:** Tricuspid annular displacement, Idiopathic scoliosis, Right ventricular function, Children and adolescents

## Abstract

**Background:**

Although it is speculated that scoliosis may induce cardiac dysfunction, there is no report about evaluation of cardiac function, especially right cardiac function in patients with scoliosis. Therefore, we evaluated right ventricular function in idiopathic scoliotic patients with mild to severe curves and compared them with healthy children and adolescents matched in age, then explored relationship between scoliosis and right ventricular function.

**Methods:**

Thirty-seven patients diagnosed with idiopathic scoliosis with a mean age of 16y/o (range, 8-25y/o) and an average spine curve of 77.5°Cobb (range, 30-157°) were studied by echocardiography. TAD was obtained using M-mode echocardiography. Similar examination was performed in a control group of 17 healthy individuals in matched-age. According to the different curve degree, all patients were divided into 3 groups (mild, moderate and severe). Comparison was done among the groups and the relationship between TAD and spine curve of Cobb was analyzed.

**Results:**

Patients with severe scoliosis showed depressed TAD. There was good correlation between TAD and spine curve of Cobb.

**Conclusions:**

Patients with severe scoliosis showed a significant lower right ventricular systolic function.

## Background

Scoliosis, especially idiopathic scoliosis is not uncommon in China. A number of studies [1,2] have reported that the deformity would affect the chest leading to respiratory dysfunction, which strongly relates to the morbidity and mortality. Some authors [3] have found that abnormalities of cardiac structure may occur in these patients. However, few studies [4] of the effect of scoliosis on heart function, especially right ventricular (RV) function have been published.

To patients with severe scoliosis, surgery is the only final treatment. Without surgery, paralysis or severe respiratory and heart failure may occur. It is very important to evaluate heart function before surgery, especially in severe scoliosis cases. If patients do have severe heart dysfunction, many troubles can be encountered during the surgery, including tachycardia, hypotension, oliguria and even cardiac arrest. In our country, the intensive care of these patients during and after surgery is the duty of not only the anesthetists but also the surgeons. If the surgeons do know the heart function well, some measures can be taken or drugs can be used to care and maintain the steady situations, and some surgical complications can be decreased in severity or even be avoided.

Now the assessment of left ventricular function is well-known, while due to the crescent shape and complex morphology of the right ventricle, precise assessment of RV function is difficult [5,6]. Recently, the tricuspid annular displacement (TAD) measured by echocardiography has been thought to be a useful way to evaluate RV function [7]. It can be obtained easily and used conveniently. We think TAD is an important and suitable measurement assessing RV function for patients with scoliosis.

The purpose of our study is to assess the possible effects of idiopathic scoliosis on RV function in children and adolescents patients. Also some mentions and advices can be given to surgeons treating scoliosis.

## Methods

### Patients, inclusion and exclusion criteria

From July 2009 to May 2012, a total of 37 patients with idiopathic scoliosis were studied in the Scoliosis Centre of the First Affiliated Hospital of Sun Yat-sen University. The types of the curves according to Lenke classification included the followings, Lenke 1AN (16 cases), Lenke 1A + (3 cases), Lenke 1A- (4 cases), Lenke 2+ (3 cases), Lenke 3AN (2 cases), Lenke 3+ (2 cases), Lenke 4+ (2 cases), Lenke 4 N (4 cases), Lenke 4- (1 cases). Besides 5 patients (Cobb’s angle < 40°), all of the 32 patients have undergone surgery in our hospital. According to the different curve degree, all patients were divided into 3 groups (mild, moderate and severe) .17 healthy individuals matched by gender and age participated in our study as a control group. The healthy subjects were volunteers who underwent regular student medical examinations or visited doctor because of physiological murmur.

Thus, four groups were formed: group 1 (patients with mild idiopathic scoliosis), group 2 (patients with moderate idiopathic scoliosis), group 3 (patients with severe idiopathic scoliosis), group 4 (control group). All of the groups were clinically assessed to exclude cases with cardiopulmonary diseases, such as asthma, cardiac structure abnormalities. Patients with scoliosis associated with Marfan’s syndrome, neurofibromatosis, skeletal dysplasia, tuberculosis or other secondary causes of scoliosis were also excluded from the study.

### Spinal deformity

All of the patients were examined by spinal X-ray, CT and MRI. Diagnosis was made by the spine surgeons according to the criteria of Scoliosis Research Society. Spinal deformity was measured from anterior-posterior and lateral views on the standing position. Spine curves were assessed using Cobb’s method. Then according to the Cobb’s angle, the scoliotic patients were divided into three groups (the above groups): mild scoliosis group (< 45°), moderate scoliosis group (45°~80°), severe scoliosis group (> 80°).

### Echocardiographic parameters

All patients and healthy subjects were examined on a GE Vivid 7 ultrasonograph (GE, Field, CT, USA) with appropriate use of probe, power, zoom and gain controls. All the echo images were performed and analyzed by the same doctor (the first author). During examination, electrocardiogram was recorded.

Firstly, the basic parameters of right ventricle were measured, including the diameter of right ventricle, right ventricular out tract (RVOT) and pulmonary artery (PA). Then, from the apical 4-chamber view, right ventricular ejection fraction (RVEF) was measured by Simpson’s method. This method is to measure end-diastolic and end-systolic volume of right ventricle, then RVEF can be calculated.

**Figure 1:**
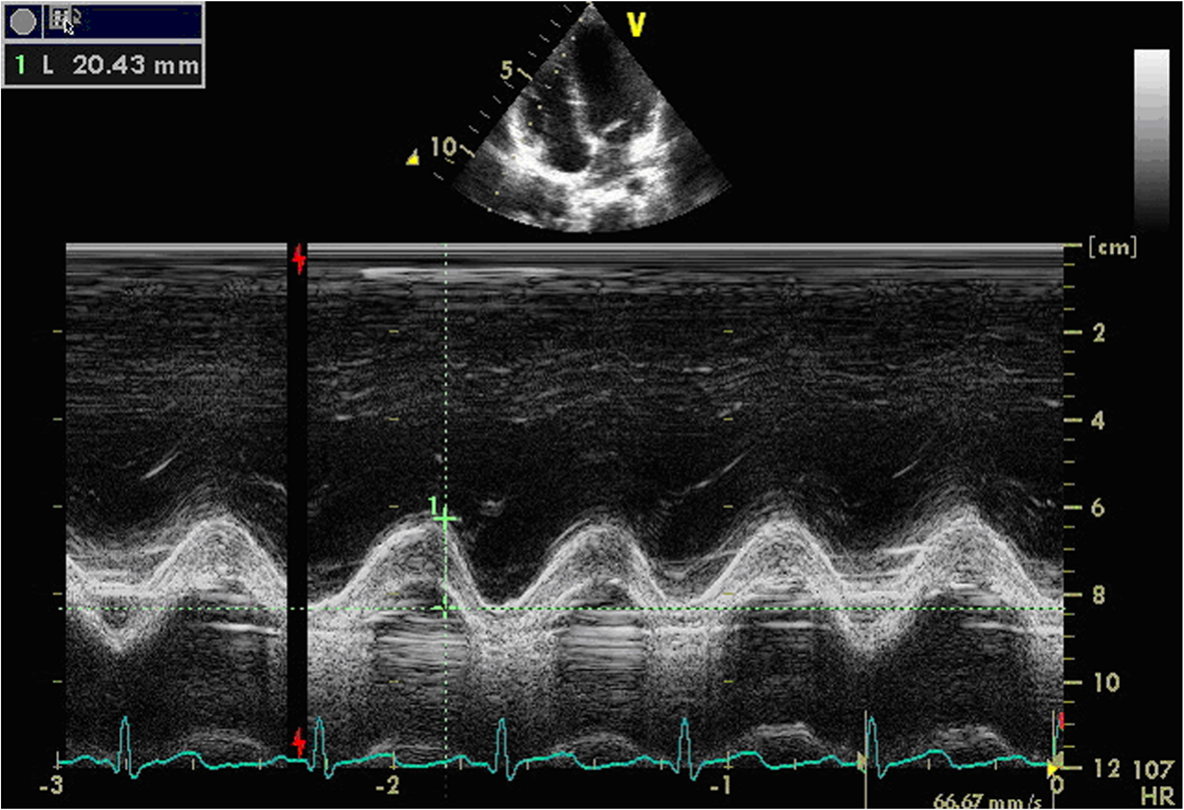
**TAD measured from the RV lateral wall (TADl).** The upper image was the apical 4-chamber view, the lower curve was the M-mode line of RV lateral wall. TADl was the distance measured form the highest to the lowest of the line (in the above image).

**Figure 2:**
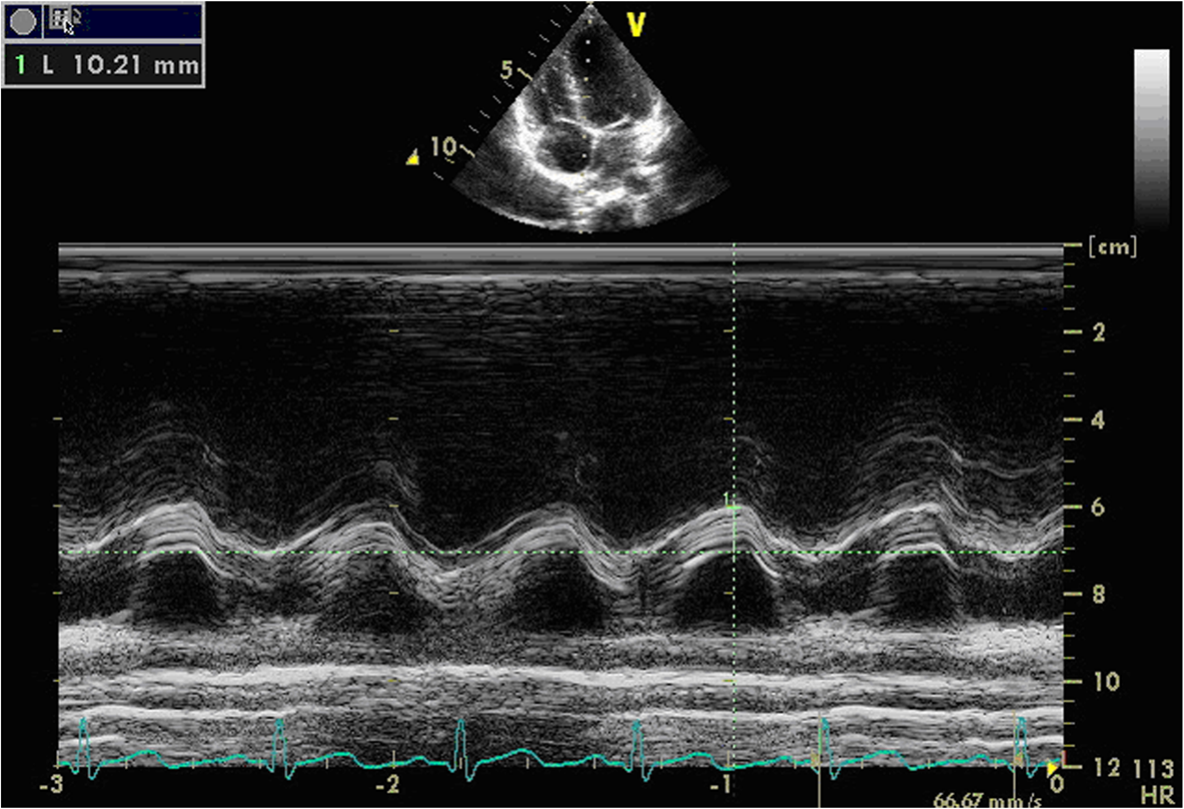
**TAD measured from the interventricular septum (TADs).** The upper image was the apical 4-chamber view, the lower curve was the M-mode line of the interventricular septum. TADs was the distance measured from the highest to the lowest of the line (in the above image).

TAD: on the active standard apical 4-chamber view, start M-mode echocardiography, put the sample line through the lateral and medial portion of the tricuspid annulus. The target is the junction of the RV lateral wall and interventricular septum with the base of the tricuspid leaflets, which delineates the RV atrioventricular plane. Then two M-mode lines were formed. TAD was the maximal distance between the leading edge of the highest and the lowest of the lines, which can be easily and precisely measured. We called TAD measured from the RV lateral wall as TADl (Figure 1), and from the interventricular septum as TADs (Figure 2).

In addition, many individuals (about 30%-40% healthy individuals) may have tricuspid valvular regurgitation (TVR), including the scoliotic patients and healthy controls in our study. To these subjects, the pulmonary artery systolic pressure (PASP) was calculated through TVR using echocardiography: To cases without pulmonary stenosis, the PASP is equal to the right ventricular systolic pressure (RVSP), RVSP is the sum of right atrial systolic pressure (RASP) and pressure gradient of TVR, RASP is 5-10 mmHg and the pressure gradient of TVR can be measured by echocardiography. Then the PASP can be well calculated by echocardiography.

### Other parameters

Forced vital capacity (FVC), forced expiratory volume in the first second (FEV1), were also recorded. These parameters reflected the lung function.

### Statistics

Statistical Package for Social Sciences (SPSS) software, version 17.0 (SPSS, Inc. Chicago, IL, USA) was used for statistical analysis. Continuous variables were summarized as mean ± SD. Differences in continuous variables among the three groups of patients and controls were investigated using one-factor analysis of variance. Variables comparison between two groups was analyzed using independent-samples *t* test. The statistical test in percentages was the *X*^2^ test. We performed Persons correlation and linear regression for identifying relationship between TAD and Cobb’s angle. Significance was established at a value of *P* < 0.05.

## Results

### Patients’ general information (Table 1)

**Table 1:** Patients’ general information

	**Group 1**	**Group 2**	**Group 3**	**Group 4**	***P***
	**Mild scoliosis**	**Moderate scoliosis**	**Severe scoliosis**	**Control group**	
	**(n = 9)**	**(n = 15)**	**(n = 13)**	**(n = 17)**	
	**Mean SD**	**Mean SD**	**Mean SD**	**Mean SD**	
Gender (M/F)	2/7	5/10	4/9	7/10	0.8
Age, yrs	16.09(4.01)	15.49(3.76)	16.33(3.65)	14.49(5.01)	0.65
Weight, kg	41.13(7.63)^c^	42.26(5.61)^c^	33.6(6.02)^abd^	46.4(12.16)^c^	<0.01
Cobb’s angle	37.67(6.52)^bc^	62.87(11.98)^ac^	122(22.29)^ab^		<0.01
FVC(L)	77.22(9.13)^c^	74.73(13.11)^c^	63.15(14.22)^ab^		0.02
FEV1(L)	72.78(8.39) ^c^	74.20(12.11) ^c^	58.38(11.36) ^ab^		<0.01
RV(mm)	20.27(3.14)	20.33(1.95)	20.27(3.44)	21.26(2.42)	0.69
RVOT(mm)	22.72(2.9)	21.87(3.68)	20.92(3.3)	23.29(2.94)	0.24
PA(mm)	19.78(4.27)	19.73(3.53)	20.23(2.17)	20.69(1.76)	0.75
RVEF(%)	61.11(5.16)	60.53(4.93)	61.38(6.34)	59.59(5.3)	0.82

Values as mean (SD); a, b, c, d represented *P* < 0.05 when comparing with group 1, 2, 3, 4 respectively.

There were no significant gender and age differences among the 4 groups. Weight of severe scoliotic patients was lower than other groups, showing poor nutritional situations in patients with severe scoliosis. There were significant differences between severe (group 3) and mild (group 1), moderate (group 2) scoliotic patients in basal ventilatory parameters (FVC, FEV1), these findings show that respiratory restrictions occurred in patients with severe scoliosis. There were no significant differences among the 4 groups in basal right ventricular parameters (diameters of RV, RVOT, PA and RVEF), which indicating that the right ventricular size and conventional functional parameter (RVEF) had not changed significantly.

## TAD results

**Figure 3:**
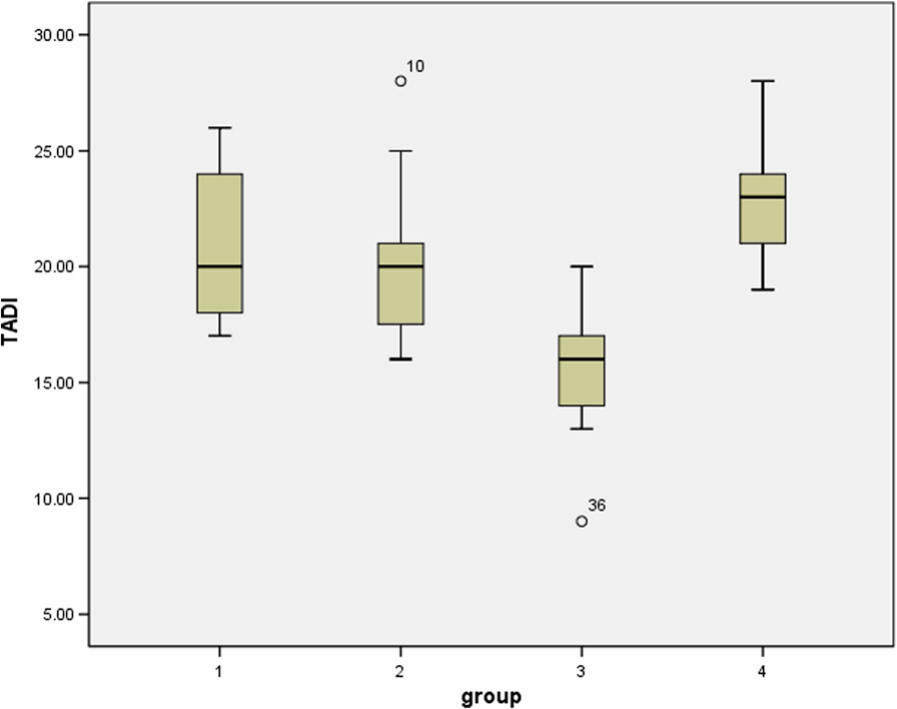
TADl in scoliotic patients and controls.

Mean TADl results in the 4 groups were as following: group 1 of 20.67 mm (SD = 3.32), group 2 of 20.13 mm (SD = 3.4), group 3 of 15.54 mm (SD = 2.73), group 4 of 22.64 mm (SD = 2.45). A statistically significant difference was found between group 3 (severe scoliosis) and other groups (Figure 3) (*P* < 0.01).

**Figure 4:**
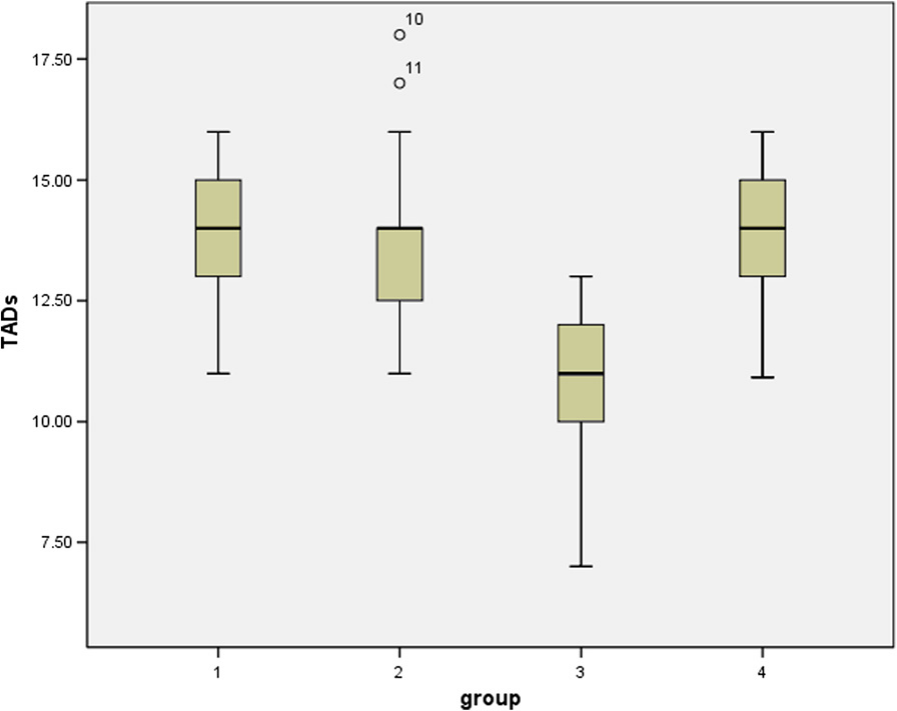
TADs in scoliotic patients and controls.

Mean TADs results in the 4 groups were as following: group 1 of 13.86 mm (SD = 1.62), group 2 of 13.73 mm (SD = 2.02), group 3 of 10.62 mm (SD = 2.06), group 4 of 14 mm (SD = 1.38). TADs of group 3 (severe scoliosis) was significantly lower than other groups (Figure 4) (*P* < 0.01).

### Correlation between TAD and Cobb’s angle

**Figure 5:**
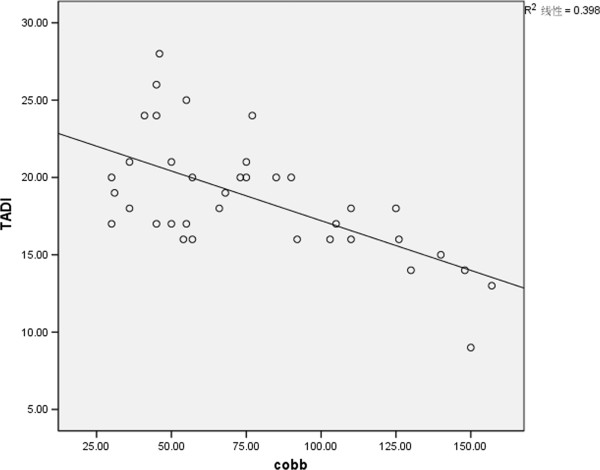
Correlation of TADl with Cobb’s angle.

**Figure 6:**
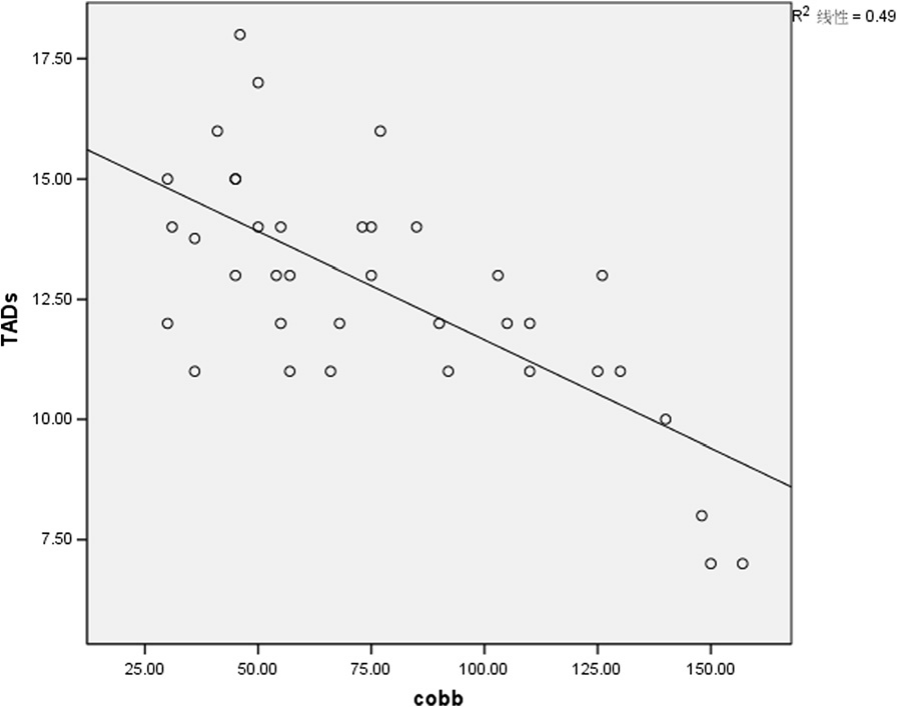
Correlation of TADs with Cobb’s angle.

TAD values (TADl and TADs) were correlated to the severity of the scoliotic curve (Cobb’s angle) . Patients with more severe curves had lower TAD values (TADl: r = −0.63, *P* < 0.01, Figure 5; TADs: r = −0.7, *P* < 0.01, Figure 6).

## PASP results

15 scoliotic patients and 6 controls had TVR. Mean PASP calculated in scoliotic patients was 20 mmHg (SD = 2.54), in controls was 18.5 mmHg (SD = 2.07). There were no significant differences (*P* = 0.17).

## Discussion

In our research, significant TAD decreases were found in 13 patients with severe idiopathic scoliosis, whose scoliotic curves exceeded 80°Cobb. Our results indicated that patients with severe idiopathic scoliosis had RV systolic functional limitations.

Surgery in severe scoliosis means a high risk of not spine dependent complications. To patients with severe scoliosis, surgeons must ensure that their heart function can endure such surgery with its extensive influence on all organs and long procedure time. The larger bloodloss and prolonged pressure on the heart (prone position during surgery) may influence the RV function. If severe RV dysfunction occurred during surgery, tachycardia and hypotension may occur, then even cardiac arrest, which can influence or interrupt surgery, or induce death. So it is very important to assess the complete cardiac function before surgery. By that the scoliosis surgeons can make appropriate decisions by assessing surgical opportunities according to the condition of the cardiac system. For example, will a patient have scoliosis surgery or drug treatment first. In our research centre, doctors who majored in pediatric cardiology, used various kinds of methods for assessing the heart function before surgery, including left and right cardiac function. This paper is a part of our study, which aimed to compare the RV function among various degree scoliotic patients and healthy subjects to determine whether differences exist.

Recently, some papers [8,9] have reported that patients with idiopathic scoliosis presented cardiorespiratory restrictions, even in patients with mild or moderate scoliosis. But these studies have evaluated the whole functions including lung and heart, and the main aspect was respiratory function. So there is a need to know the exact condition of the heart function in patients with idiopathic scoliosis. If serious RV dysfunction is found, the clinical heart function level, the size of the heart and liver, the respiratory function and the blood level of brain natriuretic peptide (BNP) should be cared first. According to the results, cardiac drugs can be decided using or not. During and after surgery, intensive care of cardiac function is necessary, cardiologists may be called.

Echocardiography is a noninvasive method for assessing cardiac function, which has been widely used in clinical work. In our research centre, every patient with idiopathic scoliosis was examined by echocardiography. Most scoliosis surgeons usually focus on the left ventricular ejection fraction (EF) and fractional shortening (FS), especially to patients with severe scoliosis. Left ventricular EF and FS are two traditional parameters for assessing left ventricular function, which have no relationship with RV function.

The importance of RV function is gradually realized by more and more doctors. Some researchers have found RV dysfunction occurred while left ventricular function was normal in patients with pulmonary hypertension [10,11]. And due to the connection of the right ventricle with pulmonary artery, the expiratory restriction usually affects the right ventricle more easily, and not so much the left one. Therefore, it is necessary to explore the condition of RV function in patients with scoliosis, which may have expiratory restriction (in our study, we found the results). But precise evaluation of RV function using echocardiography is a hard topic due to the crescent shape and complex morphology of the right ventricle.

Right ventricular ejection fraction (RVEF) measured by magnetic resonance imaging (MRI) is still thought to be the gold standard assessing the RV function. But the longer examining time and the expensive consume restricted the widely used in these patients in our country. In our research, we used improved Simpson’s method to measure RVEF. Results indicated that there were no significant differences among the groups. We think this is because patients with scoliosis have deformities of spine and chest, inducing the abnormalities of cardiac position and morphology. And the RVEF will be more easily affected by the cardiac morphology. So we think this parameter is not suitable for RV function evaluation in patients with idiopathic scoliosis.

It has been certified that the contraction of the right ventricle occurred predominantly along the longitudinal plane [12]. Thus TAD which is the systolic displacement of the tricuspid annulus toward the RV apex, closely correlates with RVEF [13] measured by MRI. Meanwhile, the measurement of TAD does not require higher equipped echo machine, RV endocardial definition or geometric assumptions. So TAD is thought to be an excellent parameter which is highly reproducible and practical, and can be used widely and conveniently in clinical work.

Some recent studies [14-17] have shown that TAD closely correlated with RVEF in a variety of populations. Sato T [18] believed that TAD had the best accuracy to assess the RV systolic function in patients with pulmonary hypertension among the echocardiography measurements. In the current study, TAD decreases have been found in patients with severe scoliosis, and our results have indicated that TAD had good negative correlation with severity of the scoliotic curve. So our study shows that RV function impaired in patients with severe scoliosis. You may concern these patients’ clinical heart function evaluation. In our study, these patients didn’t have the signs of exercise tolerance decreased, hepatomegaly, tachypnea, dyspnea, et al. We think TAD is a sensitive parameter, which finding the RV function changes before obvious clinical manifestations. To say in other words, we consider that the RV function of patients with severe scoliosis is in period of compensation, which can be easily influenced. So to these patients, surgeons have to pay attention to the volume and speed of infusion. Too large volume or rapid infusion may lead to RV dysfunction before left ventricle influenced (we have encountered these cases in our work). In our research centre, when we found TAD decreased in patients with scoliosis, we would talk to the surgeons and strengthen patients’ care during and after surgery. In some severe cases, cardiac medical treatment (cardiotonic, diuretics, et al.) may be used.

In our study, PASP evaluating by echocardiography has been compared between scoliotic patients and healthy subjects, and no significant differences have been found. We think patients with scoliosis in our study have not happened pulmonary hypertension, and TAD depression is not due to higher pulmonary pressure. These findings hint that the abnormality of the chest may induce RV dysfunction directly because of the anterior position of the right ventricle.

These results indicate that TAD is a highly sensitive and specific predictor of RV dysfunction in patients with severe idiopathic scoliosis. Thus, TAD should be incorporated into the cardiac functional assessment of patients with idiopathic scoliosis. However, TAD is a simple approach used as the first step assessment of RV systolic function. To patients with depressed TAD, we would perform further evaluation.

## Conclusions

Patients with severe scoliosis showed a significant lower right ventricular systolic function. TAD is a sensitive parameter assessing RV systolic function. Physicians treating scoliosis should pay more attention to the RV function of patients, especially the patients with severe scoliosis.

## Consent

The parents of all patients have given their consent for the report to be published.

## Competing interests

The authors declare that they have no competing interests.

## Authors’ contributions

SL carried out the studies, performed the echocardiography and drafted the manuscript. JY performed surgery of patients. YL was responsible for the cardiac assessment and treatment of the patients. LZ participated in the cardiac function assessment of the patients by echocardiography. YL and XL participated in the statistical work-out of all data. ZH participated in surgery and gave support to the final drafts on results and conclusions. HW was initiator of the study and gave advice of how to proceed and put the results in a comprehensive article. All authors read and approved the final manuscript.

## References

[B1] Takahashi S Suzuki NA Sazuma T Factors of thoracic cage deformity that affect pulmonary function in adolescent idiopathic thoracic scoliosis Spine 2007 32 106 112 10.1097/01.brs.0000251005.31255.25 17202900

[B2] Heary RF Bono CM Kumar S Bracing for scoliosis Neurosurgery 2008 3 125 130 18812914 10.1227/01.NEU.0000320387.93907.97

[B3] Ipp L Flynn P Blanco J The findings of preoperative cardiac screening studies in adolescent idiopathic scoliosis J Pediatric Orthop 2011 31 764 766 10.1097/BPO.0b013e31822f14d6 21926874

[B4] Jin-qian L Gui-xing Q Jian-xiong S A retrospective study of echocardiographic cardiac function and structure in adolescents with congenital scoliosis Chin Med J 2009 122 906 910 19493412

[B5] Guo YK Gao HL Zhang XC Accuracy and reproducibility of assessing right vent ricular function wit h 64-section multidetector row CT: Comparison with magnetic resonance imaging. Int J Cardiol 2010 139 254 262 10.1016/j.ijcard.2008.10.031 19028401

[B6] Sugeng L Mor2Avi V Weinert L Quantitative assessment of left ventricular size and function: side-by-side comparison of realtime three-dimensional echocardiography and computed tomography with magnetic resonance reference. Circulation 2006 114 654 661 10.1161/CIRCULATIONAHA.106.626143 16894035

[B7] Huques T Ducreux D Bertora D Interest of tricuspid annular displacement (TAD) in evaluation of right ventricular ejection fraction Ann Cardiol Angeiol (Paris) 2010 59 61 66 10.1016/j.ancard.2010.01.001 20356571

[B8] Ramirez M Martinez-Liorens J Bago J Significant ventilatory functional restriction in adolescents with mild or moderate scoliosis during maximal exercise tolerance test Spine 2005 30 1610 1615 10.1097/01.brs.0000169447.55556.01 16025029

[B9] Alves VL Avanzi O Objective assessment of the cardiorespiratory function of adolescents with idiopathic scoliosis through the six-minute walk test Spine 2009 34 E926 E929 10.1097/BRS.0b013e3181afd1b2 19940723

[B10] Amano H Toyoda S Arikawa T Left ventricular function in pulmonary hypertension Hear Vessel 2012 Epub ahead of print 10.1007/s00380-012-0272-3 23124961

[B11] Forfia PR Vachiery JL Echocardiography in pulmonary arterial hypertension Am J Cardiol 2012 15 16S 24S 22921027 10.1016/j.amjcard.2012.06.012

[B12] Carlsson M Uqander M Heiberg E The quantitative relationship between longitudinal and radial function in left, right, and total heart pumping in humans Am J Physiol Heart Circ Physiol 2007 293 H636 H644 10.1152/ajpheart.01376.2006 17307988

[B13] Ahmad H Mor-Avi V Lang RM Assessment of right ventricular function using echocardiographic speckle tracking of the tricuspid annular motion: comparision with cardiac magnetic resonance Echocardiography 2011 Epub ahead of print 10.1111/j.1540-8175.2011.01519.x 21967480

[B14] Papaioannou VE Stakos DA Draqoumanis CK Relation of tricuspid annular displacement and tissue Doppler imaging velocities with duration of weaning in mechanically ventilated patients with acute pulmonary edema BMC Cardiovasc Disord 2010 10 20 10.1186/1471-2261-10-20 20478065 PMC2880285

[B15] Kiotsekoglou A Sutherland GR Moggridge JC Impaired right ventricular systolic function demonstrated by reduced atrioventricular plane displacement in adults with Marfan syndrome Eur J Echocardiogr 2009 10 295 302 18801726 10.1093/ejechocard/jen239

[B16] Shah AR Grodman R Salazar MF Assessment of acute right ventricular dysfunction induced by right coronary artery occlusion using echocardiographic atrioventricular plane displacement Echocardiography 2000 17 513 519 10.1046/j.1540-8175.2000.00513.x 11000585

[B17] Hugues T Yaici K Latcu DG Usefulness of tricuspid annular displacement(TAD) to identify right ventricular dysfunction in normotensive patients with acute pulmonary embolism Ann Cardiol Angeiol 2011 60 27 32 10.1016/j.ancard.2010.12.006 21276953

[B18] Sato T Tsujino I Ohira H Validation study on the accuracy of echocardiographic measurements of right ventricular systolic function in pulmonary hypertension J Am Soc Echocardiography 2012 25 280 286 10.1016/j.echo.2011.12.012 22230250

